# Prevalence of Sexually Transmitted Infections (*Neisseria gonorrhoeae*, *Chlamydia trachomatis*, *Trichomonas vaginalis* and Human Papillomavirus) in Female Attendees of a Sexually Transmitted Diseases Clinic in Ulaanbaatar, Mongolia

**DOI:** 10.1155/S1064744901000254

**Published:** 2001

**Authors:** Suzanne M. Garland, Sepehr N. Tabrizi, Shujun Chen, Chultemsuren Byambaa, Khalzan Davaajav

**Affiliations:** 1 Department of Microbiology and Infectious Diseases The Royal Women’s Hospital 132 Grattan Street Carlton Melbourne 3053 Australia; 2 AIDS/STD Reference Center Ulaanbaatar Mongolia

## Abstract

*Background:* Epidemiological data suggest that the prevalence of syphilis, gonorrhea and trichomoniasis has increased in both urban and rural areas of Mongolia. These data are primarily substantiated by notifications of cases of clinically apparent disease in both rural and urban areas, plus laboratory diagnoses from the AIDS/STD Reference Center,Ulaanbaatar. In the past 5 years, however, there has been a marked decline in the total number of patients being screened for sexually transmitted infections (STIs). An assessment of true prevalence of STIs in a female population attending an urban sexually transmitted diseases (STD) clinic was therefore commenced.

*Methods:* Consecutivewomen attending an STD clinic in Ulaanbaatar had genital samples collected by the insertion and immediate removal of a tampon, which was then tested for the presence of *Neisseria gonorrhoeae* , *Chlamydia trachomatis*, human papillomavirus (HPV) and *Trichomonas vaginalis* , using polymerase chain reaction (PCR) amplification.

*Results:* A total of 110 women were studied (mean age 26.7 years). Overall, 58 (53%) patients had one or more pathogens identified; 43 (39%) had a single pathogen, while 15 (14%) had mixed pathogens. *C. trachomatis* was found in 15 (14%), *N. gonorrhoeae* in 12 (11%), *T. vaginalis* in nine (8%) and HPV in 39 (36%). Among the 39 HPV-positive patients, oncogenic genotypes (16, 18, 31, 33, 35, 39, 45, 51, 52) were found in 17 (44%) patients.

*Conclusions:* Sexually transmitted infections as defined by PCR were common, and found in 53%of female attendees of an urban STD clinic in Mongolia. As infections with conventional STIs increase the risk of human immunodeficiency virus (HIV) transmission, it is imperative that strategies be introduced to reduce the prevalence of STIs. Furthermore, detection of oncogenic HPV was common, indicating that it is vital that a strategy to reduce cervical cancer such as a pre-cancer cervical cytology screening program also be introduced.


### Prevalence of sexually transmitted infections (*Neisseria gonorrhoeae*, *Chlamydia trachomatis*, *Trichomonas vaginalis* and human papillomavirus) in female attendees of a sexually transmitted diseases clinic in Ulaanbaatar, Mongolia

**Suzanne M. Garland[1], Sepehr N. Tabrizi[1], Shujun Chen[1], Chultemsuren Byambaa[2] and Khalzan Davaajav[2]**

[1]*Department of Microbiology and Infectious Diseases, The Royal Women's Hospital, Melbourne, Australia*
[2]*AIDS/STD Reference Center, Ulaanbaatar, Mongolia*

***Background:*** Epidemiological data suggest that the prevalence of syphilis, gonorrhea and trichomoniasis has increased in both urban and rural areas of Mongolia. These data are primarily substantiated by notifications of cases of clinically apparent disease in both rural and urban areas, plus laboratory diagnoses from the AIDS/STD Reference Center, Ulaanbaatar. In the past 5 years, however, there has been a marked decline in the total number of patients being screened for sexually transmitted infections (STIs). An assessment of true prevalence of STIs in a female population attending an urban sexually transmitted diseases (STD) clinic was therefore commenced.
***Methods:*** Consecutive women attending an STD clinic in Ulaanbaatar had genital samples collected by the insertion and immediate removal of a tampon, which was then tested for the presence of *Neisseria gonorrhoeae*, *Chlamydia trachomatis*, human papillomavirus (HPV) and *Trichomonas vaginalis*, using polymerase chain reaction (PCR) amplification.
***Results:*** A total of 110 women were studied (mean age 26.7 years). Overall, 58 (53%) patients had one or more pathogens identified; 43 (39%) had a single pathogen, while 15 (14%) had mixed pathogens. *C. trachomatis* was found in 15 (14%), *N. gonorrhoeae* in 12 (11%), *T. vaginalis* in nine (8%) and HPV in 39 (36%). Among the 39 HPV-positive patients, oncogenic genotypes (16, 18, 31, 33, 35, 39, 45, 51, 52) were found in 17 (44%) patients.
***Conclusions:*** Sexually transmitted infections as defined by PCR were common, and found in 53% of female attendees of an urban STD clinic in Mongolia. As infections with conventional STIs increase the risk of human immunodeficiency virus (HIV) transmission, it is imperative that strategies be introduced to reduce the prevalence of STIs. Furthermore, detection of oncogenic HPV was common, indicating that it is vital that a strategy to reduce cervical cancer such as a pre-cancer cervical cytology screening program also be introduced.

Key words: SEXUALLY TRANSMITTED INFECTIONS; FEMALE; MONGOLIA; POLYMERASE CHAIN REACTION; *C. TRACHOMATIS*; *T. VAGINALIS*; *N. GONORRHOEAE*; HUMAN PAPILLOMAVIRUS

Mongolia is a sparsely populated (2.3 million) country, with 600 000 people being concentrated in the capital city of Ulaanbaatar, the only large urban area. People within the rural area (as well as 40% of those living in the city) live in gers (also called yurts), and a nomadic lifestyle is still

Correspondence to: Suzanne M. Garland, Department of Microbiology and Infectious Diseases, The Royal Women's Hospital, 132 Grattan Street, Carlton, Melbourne 3053, Australia prevalent. In the past decade there have been several changes in government, with independence from the former Soviet Union occurring in September 1990. With the introduction of privatization, travel abroad and loss of Soviet aid, there has been an increase in unemployment, alcoholism and prostitution. Epidemiological data collected by the AIDS/STD Reference Center, Ulaanbaatar show a general increase in sexually transmissible diseases, despite a marked decline in total screening (1983: gonorrhea 51 per 100 000 population, 1995: 142 per 100 000; trichomoniasis 47 per 100 000 vs. 155 per 100 000, respectively, for the same years; and syphilis 18 per 100 000 in 1993 vs. 32 per 100 000 in 1995)[1,2]. These data are largely obtained from notified clinically diagnosed cases of infection, plus laboratory diagnoses from clinically suspected cases as sent to the Reference Center in Ulaanbaatar. Resources for definitive diagnoses of various sexually transmitted infections (STIs), especially in rural areas, are very limited.

We have previously shown that patient-administered tampons as a genital sampling technique for detection of STIs by polymerase chain reaction (PCR) technology is an effective and sensitive method for determining STI prevalence, particularly when adapted to populations in remote areas[3–5]. Therefore, this methodology was adapted to determine the prevalence of various STIs in female attendees of a sexually transmitted diseases (STD) clinic in Ulaanbaatar, Mongolia.

### METHODS

### Study population

Consecutive women who attended the STD clinic at the AIDS/STD Center, Ulaanbaatar, Mongolia were invited to take part in the study. Women may have presented for a routine STI screen or for the investigation of symptomatic disease.

### Specimen collection

Prior to vaginal speculum examination, and after consent was obtained from each woman, a tampon was inserted into the vagina, then immediately removed and placed in PCR transport buffer (0.14 mol/l NaCl, 3 mmol/l KCl, 10 mmol/l $Na_2HPO_4$, 2 mmol/l $KH_2PO_4$). They were then sent to the Molecular Laboratory of the Microbiology Department, The Royal Women's Hospital, Melbourne, Australia for processing. Samples were transported by air with a travel time of 7 days. Tampons were processed by initially dislodging cells by squeezing, and subsequently pelleting by centrifugation[3]. DNA was extracted from 20-μl aliquots of tampon cell pellets using a QIAamp DNA Purification Kit (Qiagen Inc., Chatsworth, CA as per the manufacturer's instructions.

### Polymerase chain reaction

For human papillomavirus (HPV), each PCR reaction consisted of a 20-μl aliquot of extracted DNA (i.e. one-tenth of the total eluted DNA), 1 × reaction buffer (50 mmol/l KCl, 10 mmol/l Tris pH 8.3), 200 μmol/l each of deoxyadenosine triphosphate, deoxyguanosine triphosphate and deoxycytidine triphosphate (dATP, dGTP and dCTP) and 190 μmol/l deoxythymidine triphosphate (dTTP), 10 μmol/l digoxigenin–deoxyuridine triphosphate (dUTP) (Boehringer Mannheim, Mannheim, Germany), 4 mmol/l $MgCl_2$, 50 pmol of L1 consensus primers MY09–MY11[6], 5 pmol of β-globin primers GH20–PCO4[7] and 2.5 units of AmpliTaq-Gold polymerase[8] (Perkin Elmer-Cetus, Norfolk, NJ) in a total of 50 μl. The addition of digoxigenin–dUTP allowed incorporation of digoxigenin in amplicons during the amplification process.

β-Globin primers, which simultaneously amplify a human β-globin product of 260 bp, were included as a positive internal control. Generation of an amplicon by this primer indicated the presence of adequate amplifiable DNA.

The SiHa cell line (one copy of integrated HPV 16 per cell) and HEL cell line (human embryonic lung, containing no HPV DNA) were used as positive and negative controls, respectively. Specimen contamination and carry-over were prevented by using positive displacement pipettes, prior aliquoting of all reagents, and performing pre- and post-PCR steps in different rooms specifically allocated for PCR.

PCR products for the detection of HPV were analyzed by a PCR enzyme-linked immunosorbent assay (ELISA)-based detection system

(Boehringer, Mannheim). A biotinylated-L1 generic probe was produced by amplification as described by Bauer and colleagues[9]. The double-stranded 400-bp probe was denatured in 50 mmol/l NaOH, and subsequently added to a 5-μl aliquot of amplification product. Following a 10-min incubation, the denatured amplicon–probe mixture was added to Streptavidin-coated microtiter wells containing hybridization buffer supplied with the PCR ELISA kit. After a 6-h hybridization at 55°C the microtiter wells were washed, and detection of specific hybrids determined at an absorbance of 405 μm (reference filter 492 μm); the extinction value of the blank was subtracted as per the manufacturer's instructions. Positive samples were determined by the cut-off value as described above. All HPV-positive samples were typed by probing with the PCR ELISA system using type-specific, biotin-labeled, nested oligonucleotide probes for HPV 6, 11, 16, 18, 31, 33, 35, 39, 45, 51 and 52, as described by Resnick and colleagues[7]. Sub-types not belonging to this list were called 'other types'. Samples showing low-level positivity could not be typed, and were labeled as 'untypeable'.

Detection of *Chlamydia trachomatis*, *Trichomonas vaginalis* and *Neisseria gonorrhoeae* amplicons was performed as described previously[5].

### RESULTS

All 110 women invited to participate were enrolled in the study. The mean age of patients was 26.7 years. Overall, 58 (53%) women had an STI pathogen detected (Table 1), and in 43 of these (74%) only a single pathogen was detected. There was no significant difference in mean age of these women, compared with those without a detectable pathogen.

With the exclusion of those with HPV alone, 37% (30/82) had *C. trachomatis*, *N. gonorrhoeae* and/or *T. vaginalis*. Overall, for the population studied, *C. trachomatis* was detected in 15 (14%), *N. gonorrhoeae* in 12 (11%) and *T. vaginalis* in nine (8%) women (Table 1).

In total, HPV was detected in 39/110 (36%) women. HPV genotyping showed detection of oncogenic HPV 16 or 18 in nine (23%); HPV 31, 33, 35 or 39 in four (10.3%); and HPV 45, 51 or 52 in four (10.3%) patients. Overall, oncogenic HPV was detected in 44% (17/39) of HPV-positive women, with more than one genotype present in four women. Low-risk HPV 6 and 11 were detected in 18% (7/39) of HPV-positive patients. Clinically, three of these patients had condyloma accuminata, and all were HPV 6- or 11-positive.

### DISCUSSION

Sexually transmitted infections *N. gonorrhoeae*, *C. trachomatis*, *T. vaginalis* and HPV, as defined by PCR, were common, and found in 53% of female attendees of an urban STD clinic in Mongolia. Many (74%) were single-pathogen infections. With the exclusion of HPV, infections with gonorrhea, chlamydia and trichomonas collectively affected 37% of the population studied. This is a high rate, particularly when it is considered that conventional STIs increase the risk of human immunodeficiency virus (HIV) transmission[10]. These findings are consistent with those recently reported in a study using conventional diagnostic methods, in a similar patient population[2]. Therefore, it is imperative that effective strategies be introduced in Mongolia to reduce the overall prevalence of STIs. Currently, it is reported that since HIV testing was introduced in Mongolia (1987), only one case of HIV has been recognized[1]

**Table 1** Pathogens found in genital samples of women attendees at sexually transmitted diseases (STD) clinic, Ulaanbaatar

| | *Alone* (*n*) | *Mixed with ≥1 other pathogen(s)* (*n*) | *Total* (*n*) |
|---|---|---|---|
| *Chlamydia trachomatis* | 6 | 9 | 15 (14%) |
| *Neisseria gonorrhoeae* | 5 | 7 | 12 (11%) |
| *Trichomonas vaginalis* | 4 | 5 | 9 (8%) |
| Human papillomavirus | 28 | 11 | 39 (36%) |
| Any pathogen | 43 | 15 | 58 (53%) |
| No pathogen detected | NA | NA | 52 (47%) |

NA, not applicable

(and personal observation by C. Byambaa). However, HIV screening of attendees at STI clinics and other high-risk behavior groups has not been universally offered to such patients, and hence the true incidence may be greater. With this relatively high prevalence of bacterial and parasitic STIs in attendees at an urban STD clinic, a potentially explosive situation exists with respect to HIV transmission, should more cases exist in the community. With the recent greater access to travel, Mongolians are visiting countries where HIV is endemic; also, with the influx of foreign travelers and businessfolk, the potential for spread is enhanced. Strategies to reduce STIs in general and those which are culturally sensitive are vital.

Amplification assays such as PCR, when used with minimal or noninvasive patient-collected samples (tampon or first-voided urine), can revolutionize STI testing, particularly for those living in remote geographical areas [5,11,12]. Such assays have the advantage of the stability of nucleic acid in the field, compared with conventional assays which often suffer from transport delays and conditions. Hence, despite the complexities of performing amplification assays, patient-collected samples can be transported to distant sites for processing.

In addition to the conventional STIs of *N. gonorrhoeae*, *C. trachomatis* and *T. vaginalis*, human papillomavirus (HPV) was commonly found (36%), with oncogenic genotypes being present in 44% of positives. These findings underscore the importance of a cervical cancer prevention strategy, such as a Papanicolaou pre-cancer cervical cytology screening program, which is soon to be introduced.

### REFERENCES

1. Purevdawa E, Moon TD, Baigalmaa C, *et al.* Rise in sexually transmitted diseases during democratization and economic crisis in Mongolia. *Int J STD AIDS* 1997;8:398–401
2. Schwebke JR, Aira T, Jordan N, *et al.* Sexually transmitted disease in Ulaanbaatar, Mongolia. *Int J STD AIDS* 1998;9:354–8
3. Fairley CK, Chen S, Tabrizi SN, *et al.* A novel method for the assessment of human papillomavirus infection. *J Infect Dis* 1992;165:1103–6
4. Tabrizi SN, Chen S, Borg AJ, *et al.* Patient-administered tampon-collected genital cells in the assessment of *Chlamydia trachomatis* infection using polymerase chain reaction. *Sex Transm Dis* 1996; 23:494–7
5. Tabrizi SN, Paterson B, Fairley CK, *et al.* A self-administered technique for the detection of sexually transmitted diseases in remote communities. *J Infect Dis* 1997;176:289–92
6. Manos MM, Ting Y, Wright DK, *et al.* The use of polymerase chain reaction amplification for the detection of genital human papillomaviruses. *Cancer Cells* 1989; 7:209–14
7. Resnick RM, Cornelissen MT, Wright DK, *et al.* Detection and typing of human papillomavirus in archival cervical cancer specimens by DNA amplification with consensus primers. *J Natl Cancer Inst* 1990;82:1477–84
8. Lawyer FC, Stoffel S, Saiki RK, *et al.* Isolation, characterization, and expression in *E. coli* of the DNA polymerase gene from the extreme thermophile *Thermus aquaticus*. *J Biol Chem* 1989;264: 6427–37
9. Bauer HM, Yi Ting MS, Greer CE, *et al.* Genital human papillomavirus infection in female university students as determined by a PCR-based method. *J Am Med Assoc* 1991;265:472–7
10. Grosskurth H, Mosha F, Todd J, *et al.* Impact of improved treatment of sexually transmitted diseases on HIV infection in rural Tanzania: randomised controlled trial. *Lancet* 1995;346:530–6
11. Tabrizi SN, Paterson BA, Fairley CK, *et al.* Comparison of tampon and urine as self-administered methods of specimen collection in detection of *Chlamydia trachomatis*, *Neisseria gonorrhoeae* and *Trichomonas vaginalis* in women. *Int J STD AIDS* 1998;9:347–9
12. Moscicki AB. Comparison between methods for human papillomavirus DNA testing: a model for self-testing in young women. *J Infect Dis* 1993; 167:723–5

Received 4/23/01; Accepted 5/28/01
